# Social Determinants of Hypertension

**DOI:** 10.5935/abc.20190220

**Published:** 2019-10

**Authors:** José Geraldo Mill

**Affiliations:** 1 Departamento de Ciências Fisiológicas do Centro de Ciências da Saúde - Universidade Federal do Espírito Santo, Vitória, ES - Brazil; 2 Hospital Universitário Cassiano Antônio Moraes - Universidade Federal do Espírito Santo, Vitória, ES - Brazil

**Keywords:** Hypertension, Hypertension/prevention and control, Prevalence, Risk Factors, Obesity, Diabetes Mellitus, Epidemiology

Hypertension (HT) is a disease with high prevalence in adults and is generally referred to as a 'complex disease'. This term has been used to indicate the diversity of factors that contribute to its onset.^1,2^

Studies in populations, twins, and families estimate that the impact of genetic background on the onset of HT ranges from 34% to 64%.^3^ However, pressure regulation depends on a multiplicity of organs, systems, and mechanisms, which is why a large number of genes affect individual values. As a result, genetic tests are still largely ineffective as predictors of HT, since monogenic inheritance of this disease is rare.^4^ Non-genetic factors are also numerous and are linked to lifestyle (nutrition, physical activity, alcohol and tobacco consumption, among others) or to the presence of conditions connected with a chronic inflammatory state, such as obesity and insulin resistance. In the presence of these factors, blood pressure increase with age is faster, leading to, at a given moment, blood pressure levels indicative of the presence of the disease. It is important to point out that the cutoffs that separate the states of 'normotension' and 'hypertension' are statistical, being suitable for use with populations, and potentially unsuitable in assessing individuals,^5^ as the disease may be present in a subclinical state, i.e., even before reaching diagnostic blood pressure levels obtained in epidemiological studies. Therefore, given the difficulty of using genetic data, disease prevention should be undertaken by identifying risk factors that contribute to raising blood pressure. In this context, epidemiological knowledge about specific populations is an essential tool for handling the disease.

Despite its high impact on morbidity and mortality, and on economic and social costs, the epidemiology of HT and its determinants are still poorly known for the Brazilian population. Only in recent years has a robust and nationwide study been conducted in this area. The large territorial extent and the racial and cultural diversity of the Brazilian population also require regional studies. The National Health Survey (*PNS*) conducted in 2013 by the Ministry of Health with support from the *IBGE* [Brazilian Geography and Statistics Institute) in a representative and robust sample (N > 60 thousand) of the Brazilian adult population, showed a self-reported prevalence of HT of 21.4%, more frequent in women (24.2%) than men (18.3%). Upon changing the diagnostic criterion, considering those with blood pressure measured at home ≥ 140/90 mmHg, or those taking antihypertensive drugs, as individuals with HT, the prevalence rose to 32.3%, with a higher prevalence in men.^6^ The study also showed differences between regions, with lower prevalences seen in the North and Northeast and higher ones in the South and Southeast. The disease was also less frequent in rural residents. Part of the regional differences may stem from different race/color compositions. Indigenous peoples apparently have lower blood pressure readings^7^ and this may translate into a lower impact of the disease on populations with a greater presence of the indigenous trait, such as in the Northern region. Regional differences may also stem from the uneven distribution of general factors that affect blood pressure regulation, such as high salt intake, body fat accumulation, physical inactivity, alcohol abuse, and insulin resistance. The large territorial extent and cultural diversity may contribute to the non-uniform distribution of these factors and, consequently, the variability in the distribution of HT and other chronic diseases. More recently, the role of socioeconomic variables in the emergence, progress, and outcomes related to blood pressure is being viewed with increasing importance. Large studies, such as the Longitudinal Study of Adult Health (*ELSA-Brasil*), show the impact of low education and income levels on increased blood pressure and on the prevalence of the disease.^8^ These data indicate that the Brazilian population segment living in more unfavorable conditions is more subject to the impact of the disease. And this has important consequences for addressing this health problem.

In this issue of the *Arquivos Brasileiros de Cardiologia* [Brazilian Cardiology Archives], Santiago et al.^8^ publish data on a population-based study aimed at identifying the characteristics and prevalence of HT in the adult population (20-59 years) residing in the semiarid region of Pernambuco, in the Northeastern region of Brazil.^8^ For this purpose, a representative sample of urban and rural households was selected by drawing from census tracts from three municipalities. The study showed that the overall prevalence of HT was 27.4%, with a predominance in males. Despite not having the statistical power for more detailed analyses of subgroups, it is clear from the data that the disease affects, with greater impact, the population segments with lower education and income, two variables that are represented in the socioeconomic classification of the households. The association between the presence of the disease and low education is impressive. While in the segment with higher education the presence of HT was found in 15.4% of the individuals, in the lowest segment the number increased to 44.6%, i.e., the probability of the disease being found was almost 3 times higher in the population segment with a low education level. Considering that education and income are two collinear variables in the Brazilian population, in the multivariate analysis model, schooling level dropped out of the modeling, leaving only the socioeconomic level as an independent predictor of the disease's presence. However, according to the Brazilian standard, both education and income enter into the socioeconomic classification model.

What still needs to be further investigated is the mediation between socioeconomic variables (education and income) and blood pressure. The *ELSA-Brasil* provided some clues on this.^9^ Participants of African ancestry (Blacks and Browns) present higher blood pressure and higher blood pressure increase with age, thus predisposing to the onset of HT in adulthood. It is not known, however, whether this difference arises from birth or occurs later. Our research group has been seeking answers by studying children and adolescents of different races/color. We have shown that pre-pubertal students have equal blood pressure values, regardless of race/color.^10^ The differences, therefore, appear later in adolescence or, more likely, in adulthood. Psychosocial stress could constitute an important factor in increased pressure with age and, therefore, in the onset of HT.^11^ This could explain, albeit in part, the inverse relationship between education/income and HT prevalence. Individuals at the bottom of the social pyramid would live in greater uncertainty regarding their future. The struggle for survival is greater and the social support network related to adverse events in life (unemployment, adverse weather events such as prolonged drought in rural backlands) is less at the base of the pyramid, and this would determine a higher intensity allostatic load on these individuals (increased sympathetic activity, activation of the hypothalamic-adrenal-cortisol axis, attenuation of vagal function) contributing to a faster blood pressure increase over time and contributing to the earlier onset of hypertensive disease. Even without yet understanding where the initial deregulation that would lead to essential HT would be, this chain of events could explain the findings described by Santiago et al.^8^ and other authors. This reasoning could explain, in theory, the small decrease in the prevalence of HT in Brazil described by Picon et al.^12^ in a meta-analysis based on population-based studies with direct blood pressure measurement.^12^ It is noteworthy that in this meta-analysis almost all studies were done in cities in the South and Southeast regions of Brazil, where the population's educational level has been improving in recent decades.

Regardless of the mechanism, the data described for the Brazilian population, showing an inverse relationship between education level and HT, pose an additional challenge in addressing the problem. Once diagnosed, the disease must be treated. At this stage, the adoption of healthy lifestyle habits is mandatory in relation to diet (rich in whole grains, fresh fruits and vegetables), physical activity, and quitting smoking and alcohol abuse. If such measures are insufficient for pressure normalization, then medication use enters as an effective measure. However, various factors contribute to the fact that both the adoption of healthy habits, as well as the use of medications, is more difficult for individuals in lower socioeconomic segments. Therefore, those who are most affected by the disease will have less conditions to treat it. Medications, although effective, must be used correctly, as their improper use can do more harm than good. Considering that the gateway to the diagnosis and treatment of HT in our country is the primary care sector, represented by the Primary Care Units, it is essential to engage all health teams, involving doctors, nurses, nutritionists, etc., so that the effectiveness of treatments for the hypertensive population becomes as homogeneous as possible, that is, regardless of socioeconomic factors. On the other hand, the data point to a fact of great significance. Improved education brings about health benefits in general and, particularly, for addressing chronic diseases, such as HT. Investments in education affects favorably the population health.
